# Supplementary material for: In vivo mitochondrial ATP production is improved in older adult skeletal muscle after a single dose of elamipretide in a randomized trial
Source: PLoS One. 2021 Jul 15;16(7):e0253849. doi: 10.1371/journal.pone.0253849 (PMC8282018; doi:10.1371/journal.pone.0253849)
Supplement: S1 Protocol — Approved protocol for this clinical trial, including a description of the primary and secondary endpoints, inclusion and exclusion criteria, and statistical plan can be found in the Supplemental Information. (PDF) [file pone.0253849.s002.pdf]

Clinical Study Protocol

**Study Title:**	A Phase 2a Randomized, Double-Blind, Placebo-Controlled Study to Evaluate the Impact of a Single Intravenous Dose of MTP-131 (Bendavia™) on Skeletal Muscle Function in the Elderly
**Stealth BioTherapeutics Study Number:**	SPITM-201
**Document Status:**	FINAL
**Date of Original Protocol**	19 June 2014
**Date of Protocol Amendment 1.0**	17 June 2015
**Date of Protocol Amendment 1.1**	21 January 2016
**IND Number:**	105,942
**Sponsor:**	Stealth BioTherapeutics Inc.<br>275 Grove Street, Suite 3-107<br>Newton, MA 02466
**Sponsor Medical Monitor:**	James Carr, PharmD
**Protocol Version Number:**	2.1

Emergency Contact Information

Sponsor Contact<br>(Medical Monitor)	James Carr, PharmD<br>275 Grove Street; Suite 3-107<br>Newton, MA 02466<br>+1 (617) 600-6888<br>jim.carr@stealthbt.com
Principal Investigator<br>24-hour Emergency Contact	Baback Roshanravan, MD<br>The University of Washington Medical Center<br>Box 359606, 908 Jefferson Street, 3rd Floor, NJB353<br>Seattle, WA 98104<br>+1 (206) 616-8574
Coordinating Investigator	Kevin Conley, PhD<br>The University of Washington Medical Center<br>Box 357115, 1959 NE Pacific St.<br>Seattle, WA 98195<br>+1 (206) 543-3763

1. SYNOPSIS

**Name of Sponsor/Company:**<br>Stealth BioTherapeutics Inc.	
**Name of Investigational Product:**<br>MTP-131 (Bendavia™)	
**Title of Study:** A Phase 2a Randomized, Double-Blind, Placebo-Controlled Study to Evaluate the Impact of a Single Intravenous Dose of Bendavia™ (MTP 131) On Skeletal Muscle Function in the Elderly	
**Number of Planned Subjects:**<br>Screened: 60<br>Randomized: 45<br>Completed: 40	**Phase of Development:** 2a
**Name of Principal Investigator:**<br>Baback Roshanravan, MD	
**Study Center:** This study will be conducted at 1 study center, the University of Washington Medical Center I Seattle, Washington 98195	
**Objectives:**<br>**Primary:**<br>To evaluate the effect of MTP-131, given as an intravenous (IV) infusion on hand skeletal muscle energetics and muscle performance as measured by in vivo 31 phosphorus-31 (31 P) magnetic resonance spectroscopy (MRS), in vivo optical spectra scan (OPS), and a muscle fatigue test in elderly subjects with evidence of mitochondrial dysfunction.<br>**Secondary:**<br>• To assess the safety and tolerability of a single IV infusion of MTP-131 in elderly subjects with evidence of skeletal muscle mitochondrial dysfunction.<br>• To assess plasma concentrations of MTP-131 following a single IV infusion in elderly subjects in comparison to plasma concentrations demonstrated in earlier clinical studies.	
**Study Design Overview:** This is a randomized, double-blind placebo-controlled, single-center study in male and female elderly subjects with evidence of skeletal muscle mitochondrial dysfunction. This study will utilize a single double-blind dose of either MTP-131 or Placebo and will screen a sufficient number of subjects in order to have 40 subjects complete the study. The planned duration of the study includes a Screening Period of up to 28 days, a 1 day Treatment Period and a 7 day Observation Period.	
**Diagnosis and Main Criteria for Inclusion:** Adults enrolled in this study will be ≥ 60 and ≤ 85 years-of-age, with a body mass index (BMI) between 16 and 35 kg/m2 with mitochondrial dysfunction defined as in vivo 31P MRS- and OPS determined by maximal adenosine triphosphate (ATP) synthetic rate (phosphorylation capacity per unit muscle volume [ATP]) max < 0.70 mM/sec and in vivo 31P MRS- and OPS determined mitochondrial coupling Phosphate/Oxygen (P/O) of < 1.9.	
**Investigational Product, Dosage, and Mode of Administration or Intervention:** MTP-131 given as an intravenous infusion at 0.25 mg/kg/hour and at a rate of 60 mL/hour for 2 hours.	

Planned Duration of Treatment: 1 week
Screening period: 28 days
Treatment period: 1 day
Observation period: 7 days

Reference Therapy, Dose, and Mode of Administration or Comparative Intervention: Placebo (lyophilized excipients without the active drug) given as an IV infusion at a rate of 60 mL/hour for 2 hours.

Criteria for Evaluation:

Primary endpoint

The primary efficacy endpoint is change from Baseline after study drug infusion in the maximum ATP synthetic rate (ATP_{max}).

Actual values and change from Baseline will be summarized by treatment group. The primary analysis set is the intent-to-treat (ITT) population.

Secondary endpoints

Secondary analyses to address the primary objective will include comparisons in change from Baseline between treatment groups for the following hand skeletal muscle energetics and functional properties that will be analyzed in the same manner as the primary efficacy measures:

- P/O
- Nicotinamide adenine dinucleotide (NAD) levels
- Muscle force-time-integral

Mean change from Baseline between treatment groups with respect to muscle performance measured as muscle work rate and maximum integrated force generated will be calculated from the results of the hand muscle fatigue test. Change from Baseline for the above measures will be assessed at Hour 2 (2 hours after the start of infusion or end of infusion) and Day 7, except for muscle performance that will also be assessed at Day 3. Mean change between treatment groups will be compared in an analysis of covariance (ANCOVA) framework, with Baseline as a covariate. Plasma Concentration Analyses

Plasma samples will be analyzed for MTP-131 concentrations and compared to historical values obtained in prior human pharmacokinetic studies. No formal statistical analyses will be performed.

Statistical Methods:

Safety Analysis

All reported adverse events (AEs) will be listed, but only treatment-emergent adverse events (TEAEs) will be summarized. The incidence of all TEAEs, drug relationship with TEAEs, and severity of TEAE will be summarized by treatment group. Listings will be provided for deaths, serious adverse events (SAEs), and AEs leading to discontinuation of study or study drug. Summary tables for laboratory parameters (including hematology, chemistry, and urinalysis) will include descriptive statistics for values and change from Baseline for all continuous variables for each treatment group. Subjects with laboratory data outside the normal range will be listed with abnormal values flagged. Vital signs change from Baseline and shift tables will be summarized by treatment group and by study visit. Electrocardiogram results (normal versus abnormal) and an assessment of the clinical significance of any abnormalities (in the opinion of the Investigator) will be listed for individual subjects. Intervals of PR, QRS and QTc will also be listed.

Efficacy Analysis

For this Phase 2a study, no adjustments will be made to the type 1 error to account for multiple efficacy measure comparisons. Formal statistical tests (where performed) will be 2-sided and tested at the alpha=0.05 level of significance.

In general, categorical variables will be summarized by the count (N) and percentage of subjects (%). Continuous variables will be summarized by the number of non-missing observations (N), mean, standard deviation, median, minimum, and maximum values.

All study data are to be displayed in the data listings.

Statistical analysis of this study will be the responsibility of the Sponsor or its designee.

Efficacy analyses will be conducted on the ITT population. This set includes all data from all randomized subjects receiving at least 1 dose of the investigational medicinal product (IMP) according to the treatment the subjects were assigned.

All tests of treatment effects will be conducted at a 2-sided alpha level of 0.05.

Subject disposition summaries will include the number of subjects randomized and the numbers included in the Safety and ITT populations by treatment group for all subjects. The number and percentage of subjects who complete or discontinue from the study will be summarized by reason for discontinuation for each treatment group.

Subject's age, sex, weight, height, BMI, and other demographic characteristics will be recorded and summarized by treatment group.

Medical history will be listed.

Sample Size

This study is powered to detect a difference in ATP_{max}, between treatment groups.

Previous studies in endurance training in subjects aged 65 to 80 years suggest the mean ATP_{max} of 0.54 mM/sec, with a standard deviation of 0.04 mM/sec at baseline in the pre-training group (Conley et al., 2013). A total of 20 subjects per treatment group will provide for approximately 90% power to detect difference in mean ATP_{max} of 0.063, between treatment groups, assuming that the common standard deviation is 0.06 using a two group t-test with a 0.05 two-sided significance level.

2. TABLE OF CONTENTS

1. Synopsis ... 3
2. Table of Contents ... 6
3. Abbreviations and Definitions ... 9
4. Introduction ... 11
5. Objectives ... 12
   - 5.1. Primary Objective ... 12
   - 5.2. Secondary Objective ... 12
6. Investigational Plan ... 13
   - 6.1. Summary of Study Design ... 13
   - 6.2. Discussion of Design and Control ... 13
     - 6.2.1. Study Assessments ... 14
7. Study Population ... 18
   - 7.1. Inclusion Criteria ... 18
   - 7.2. Exclusion Criteria ... 18
   - 7.3. Prohibited Medications ... 20
   - 7.4. Discontinuations ... 20
     - 7.4.1. Discontinuation of Subjects ... 20
     - 7.4.2. Discontinuation of Study Sites ... 21
     - 7.4.3. Discontinuation of the Study ... 21
8. Treatment ... 22
   - 8.1. Treatments Administered ... 22
   - 8.2. Materials and Supplies ... 22
     - 8.2.1. MTP-131 ... 22
     - 8.2.2. Placebo ... 22
   - 8.3. Method of Assignment to Treatment ... 23
   - 8.4. Selection and Timing of Doses ... 23
   - 8.5. Continued Access to Investigational Product ... 23
   - 8.6. Emergency Unblinding ... 23
   - 8.7. Concomitant Therapy ... 23
   - 8.8. Treatment Compliance ... 24
9. Efficacy, Safety Evaluations, Sample Collection and Testing, and Appropriateness of Measurements ... 25
   - 9.1. Efficacy Measures ... 25
     - 9.1.1. Skeletal Muscle Energetics ... 25
     - 9.1.2. Muscle Performance ... 25
   - 9.2. Safety Evaluations ... 25
     - 9.2.1. Adverse Events ... 26
   - 9.3. Pre-Treatment Events ... 26

9.4. Baseline Medical Conditions 26
9.4.1. Abnormal Laboratory and Other Abnormal Investigational Findings 26
9.4.2. Serious Adverse Events 27
9.4.3. Events that Do Not Meet the Definition of a Serious Adverse Event 27
9.4.4. Recording of Adverse Events 27
9.4.5. Investigator Assessments 28
9.4.6. Adverse Event Reporting Period 29
9.4.7. Serious Adverse Event Expedited Reporting 29
9.4.8. Pregnancy and In Utero Drug Exposure 30
9.4.9. Responsibilities to Regulatory Authorities, Investigators, Ethics Committees, and Ethical/Institutional Review Boards 30
9.5. Sample Collection and Testing 31
9.5.1. Samples for Study Qualification and Health Monitoring 31
9.5.2. Samples for Drug Concentration Measurements 31
9.6. Appropriateness of Measurements 31
10. Data Quality Assurance 32
10.1. Data Capture System 32
11. Sample Size and Statistical Methods 33
11.1. Determination of Sample Size 33
11.2. Statistical and Analytical Plans 33
11.2.1. General Considerations 33
11.2.2. Subject Disposition 34
11.2.3. Subject Characteristics 34
11.2.4. Concomitant Therapy 34
11.2.5. Primary Efficacy Analyses 34
11.2.6. Secondary Efficacy Analyses 34
11.2.7. Plasma Concentration Analyses 35
11.2.8. Safety Analyses 35
11.2.9. Interim Analyses 36
12. Informed Consent, Ethical Review, and Regulatory Considerations 37
12.1. Informed Consent 37
12.2. Ethical Review 37
12.3. Regulatory Considerations 37
12.3.1. Protocol Signatures 38
12.3.2. Final Report Signature 38
12.3.3. Study Monitoring 38
12.3.4. Retention of Records 38
12.3.5. Disclosure of Information 38
13. References 40
Attachment 1. Study Schedule 41

Attachment 2. Clinical Laboratory Tests 44

Attachment 3: Magnetic Resonance Spectroscopy, Optical Spectroscopy, and Muscle Fatigue Testing Protocols 45

Attachment 4. Investigator and Sponsor Agreement and Signature Page...................................... 48

3. ABBREVIATIONS AND DEFINITIONS

Term	Definition
AE	Adverse event
ATP	Adenosine triphosphate
ATP_{ase}	ATP flux in resting muscle
ATPgly	Rate of ATP synthesis by glycolysis
ATPgly/ATPcost	The fraction of ATP production by glycolysis
ATP_{max}	Maximal ATP synthetic rate (phosphorylation capacity per unit muscle
volume) ATP/O_2	Mitochondrial energy coupling
BMI	Body mass index
BP	Blood pressure
BUN	Blood urea nitrogen
CIOMS	Council for International Organizations of Medical Sciences
CK	Creatine kinase
C_{max}	Maximum plasma concentration
DBP	Diastolic blood pressure
ECG	Electrocardiogram
eCRF	electronic Case Report Form
ERB	Ethical Review Board
FDA	Food and Drug Administration
FDI	First dorsal interosseous
GCP	Good Clinical Practice
Hgb	Hemoglobin
IB	Investigator's Brochure
ICF	Informed consent form
ICH	International Conference on Harmonisation of Technical Requirements for Registration of Pharmaceuticals for Human Use
IMP	Investigational medicinal product
ITT	Intent-to-treat
IV	Intravenous
IWRS	Interactive Web-based Response System
Mb	Myoglobin
MedDRA	Medical Dictionary for Regulatory Activities
MRS	Magnetic resonance spectroscopy
MTP-131	Bendavia™
MVC	Maximum Voluntary Contraction
NAD	Nicotine adenine dinucleotide
OPS	Optical spectra scan
PCr	Phosphocreatine
PK	Pharmacokinetic
^{31}P	Phosphorus-31
P/O	Phosphate/Oxygen ratio
PT	Preferred term
RBC	Erythrocyte count
SAE	Serious adverse event
SAP	Statistical Analysis Plan

SBP	Systolic blood pressure
SOC	System organ class
SUSAR	Suspected unexpected serious adverse reactions
TEAE	Treatment-emergent adverse event
US	United States
WBC	Leukocytes
WHO-DD	World Health Organization Drug Dictionary

4. INTRODUCTION

Mitochondrial dysfunction in aging skeletal muscle is an important public health concern due to its role in exercise intolerance and skeletal muscle dysfunction (sarcopenia). Mitochondria are responsible for meeting the adenosine triphosphate (ATP) demands for sustained muscle contraction. Impaired mitochondrial function is associated with exercise intolerance and fatigue, which lead to poor quality of life, loss of independence, and age-related diseases (Rondelli et al., 2009; Smart 2011; Smart et al., 2007; Williams et al., 2007). Loss of independence results from an inability to perform activities of daily living that require sustained muscle power, such as walking, dressing, and showering, as well as increased risk of falling (Bortz 2002). Increased rates of nursing home placement and hospitalization for the elderly make the loss of skeletal muscle function with age a growing public health crisis in terms of both quality of life and economic costs to society (Janssen et al., 2004). These costs were estimated at $18 billion United States (US) dollars in 2000 and it was predicted that a 10% reduction in sarcopenia prevalence would lead to a savings of $1.4 billion in healthcare costs (adjusted to 2010 dollars).

MTP-131 for intravenous (IV) infusion (Bendavia™) is a small mitochondrial targeting peptide that appears to protect the mitochondrion from a variety of insults. Initial Phase 1 studies have shown that MTP-131 is generally well-tolerated at doses up to 0.25 mg/kg/hour when infused over 4 hours or less.

MTP-131 has been shown in animal models to correct acute muscle dysfunction induced by mechanical ventilation (Powers et al., 2011), subacute dysfunction induced by either skeletal muscle immobilization (Min et al., 2011) or burns (Righi et al., 2013), and age-induced, chronic skeletal muscle dysfunction (Siegel et al., 2013). The improvements observed in these animal models, both in muscle function and biomarker levels, can be assessed immediately following IV infusions of MTP-131.

This study will be a Phase 2a, randomized, double-blind, placebo-controlled study, enrolling subjects with previous evidence of mitochondrial dysfunction at a single site to evaluate whether the administration of MTP-131 will change both the biomarker and functional performance of age-related skeletal muscle mitochondrial dysfunction in an elderly population. Previous studies involving elderly subjects (with and without renal dysfunction) have not demonstrated population specific safety concerns.

This study will be conducted in strict compliance with the protocol, current Good Clinical Practice (GCP) and all Food and Drug Administration (FDA) guidelines.

Detailed information about the potential benefits and risks of MTP-131 may be found in the Investigator's Brochure (IB).

5. OBJECTIVES

5.1. Primary Objective

The primary objective of this study is to evaluate the effect of MTP-131, given as an IV infusion on hand skeletal muscle energetics and muscle performance as measured by in vivo phosphorus-31 (31P) magnetic resonance spectroscopy (MRS), in vivo optical spectra scan (OPS), and a muscle fatigue test (Attachment 3) in elderly subjects with evidence of mitochondrial dysfunction.

5.2. Secondary Objective

The secondary objectives of the study are to assess the safety and tolerability, of a single IV infusion of MTP-131 in elderly subjects with evidence of skeletal muscle mitochondrial dysfunction. In addition, plasma concentrations of MTP-131 following a single IV infusion in elderly subjects will be compared against historical levels determined from previous pharmacokinetic studies.

6. INVESTIGATIONAL PLAN

6.1. Summary of Study Design

This is a Phase 2a, randomized, double-blind, placebo-controlled study enrolling elderly subjects. The objective is to assess the impact of MTP-131, given as a single IV infusion of 0.25 mg/kg/hour at a rate of 60 mL/hour for 2 hours, on skeletal muscle energetics and muscle performance in elderly subjects with evidence of skeletal muscle mitochondria dysfunction. Together, skeletal muscle energetics and muscle performance comprise “skeletal muscle function.”

Hand skeletal muscle function will be determined using the following independent measurements (Attachment 3):

- A muscle fatigue test (exercise instructions) to determine the mitochondrial phosphorylation capacity (ATP_{max}) (Attachment 3)
- These 2 measurements below will provide P/O data:
  - *in vivo* ^{31}P MRS to assess key high-energy compounds (phosphocreatine and ATP) to determine mitochondrial phosphorylation
  - *in vivo* OPS to measure hemoglobin (Hgb) and myoglobin (Mb) oxygenation states and relative concentrations ([Hgb]/[Mb]) to determine mitochondrial respiration (oxygen uptake)
- A sustained hand fatigue test (Attachment 3) to determine integrated force generation and fatigue threshold of the muscle

Elderly subjects will be screened for mitochondrial dysfunction, defined as an in vivo 31P MRS- and OPS-determined $ATP_{max} < 0.70$ mM/sec and P/O < 1.9.

Subjects who meet eligibility criteria will be randomized and receive study drug within 28 days of screening, or will be considered a screen failure. Subjects may be randomized only once into the study.

6.2. Discussion of Design and Control

This is a randomized, double-blind placebo-controlled, single-center study in male and female elderly subjects with evidence of skeletal muscle mitochondrial dysfunction. This study will utilize a single double-blind dose of either MTP-131 or Placebo and will screen a sufficient number of subjects in order to have 40 subjects complete the study.

In this study, MTP-131 will be administered in order to evaluate the effect on ATP production in skeletal muscle in subjects with previous evidence of mitochondrial dysfunction. Placebo was chosen as the comparator in this study in order to differentiate the natural variability in the end point measurements from true study drug effects.

Randomization will be used in this study to avoid bias in the assignment of subjects to study treatments, to increase the likelihood that known and unknown subject attribute (e.g., demographics and Baseline characteristics) are balanced across treatment groups, and to enhance the possible validity of statistical comparisons across treatment groups. Blinded treatment will be used to reduce potential bias during data collection and evaluation of endpoints.

Only those subjects who meet protocol-specified criteria for skeletal muscle mitochondrial dysfunction will be enrolled into the study (Section 7.1).

6.2.1. *Study Assessments*

The timing of all study assessments are presented in Attachment 1 and are detailed in the following sections. A list of all clinical laboratory tests to be performed and a sampling summary is found in Attachment 2.

6.2.1.1. Screening (Visit 1/Day -28 to Day -7)

- Informed consent will be signed
- Inclusion and exclusion criteria will be reviewed
- Demographics will be recorded (age, gender, ethnicity, race)
- Medical and surgical history will be recorded
- Prior and concomitant medications will be recorded and excluded medications will be reviewed
- Physical examination (general appearance, skin, head, eyes, ears, nose, throat, neck [including thyroid], lymph nodes, chest, heart, abdomen, extremities, and nervous system)
- Weight, height, and BMI calculation
- Vital signs (heart rate and blood pressure [BP] after being supine for 5 minutes, and oral or tympanic temperature)
- Electrocardiogram (ECG; all scheduled ECGs must be performed after the subject has rested quietly for at least 5 minutes in the supine position)
- Hematology analyses(hemoglobin, hematocrit, erythrocyte count [RBC], mean cell volume, mean cell hemoglobin concentration, leukocytes [WBC], neutrophils, lymphocytes, monocytes, eosinophils, basophils, platelets)
- Chemistry analyses (sodium, potassium, total bilirubin, direct bilirubin, alkaline phosphatase, alanine aminotransferase [ALT], aspartate aminotransferase [AST], blood urea nitrogen [BUN], creatinine, uric acid, calcium, nonfasting glucose, albumin, cholesterol, creatine kinase [CK])
- Urinalysis (specific gravity, pH, protein, glucose, ketones, blood, urine leukocyte esterase)
- ^{31}P MRS: See Attachment 3 for details of the procedures
- OPS: See Attachment 3 for details of the procedures
- Hand fatigue test: See Attachment 3 for details of the procedure
- Pre-treatment adverse events ([AEs] occurring before administration of study drug)
  Note*: Adverse events that occur between the time subject signs the informed consent form and the time the subject is dosed with study drug will be summarized in the medical history eCRF and not as an AE unless the event meets the definition of an SAE. This applies to screen failures as well. For subjects who fail screening, AEs and updates (if applicable) must be recorded in the medical history eCRF until the date the subject was determined to have failed screening.*

6.2.1.2. Pre-infusion (Visit 2/Day 1)

- Subjects will be admitted to the clinical research unit
- Inclusion and exclusion criteria will be reviewed
- Prior and concomitant medications will be recorded and excluded medications will be reviewed
- Physical examination (general appearance, skin, head, eyes, ears, nose, throat, neck [including thyroid], lymph nodes, chest, heart, abdomen, extremities, and nervous system)
- Weight
- Vital signs (heart rate and BP after being supine for 5 minutes, and oral or tympanic temperature)
- ECG (all scheduled ECGs must be performed after the subject has rested quietly for at least 5 minutes in the supine position)
- Hematology analyses (hemoglobin, hematocrit, erythrocyte count [RBC], mean cell volume, mean cell hemoglobin concentration, leukocytes [WBC], neutrophils, lymphocytes, monocytes, eosinophils, basophils, platelets)
- Chemistry analyses (sodium, potassium, total bilirubin, direct bilirubin, alkaline phosphatase, alanine aminotransferase [ALT], aspartate aminotransferase [AST], blood urea nitrogen [BUN], creatinine, uric acid, calcium, nonfasting glucose, albumin, cholesterol, creatine kinase [CK])
- Blood will be drawn for PK measurements
- Urinalysis (specific gravity, pH, protein, glucose, ketones, blood, urine leukocyte esterase)
- Additional urine and blood will be collected for testing of oxidative stress biomarkers
- Additional urine and blood will be collected for testing osmolality
- Laboratory results will be reviewed and all eligibility criteria will be verified
- Subjects who continue to meet all eligibility criteria will be randomized
- Record pre-treatment AEs (AEs occurring before administration of study drug) on the medical history electronic case report form (eCRF). If serious, complete AE eCRF

6.2.1.2.1. Infusion Start (Hour 0)

- Study drug (either MTP 131 at 0.25 mg/kg/hour or Placebo) will be started at an infusion rate of 60 mL/hour for 2 hours
- AEs will be recorded

6.2.1.2.2. Hour 2; Time of Infusion End and 2 Hours after the Start of Infusion

- Vital signs (heart rate and BP after being supine for 5 minutes, and oral or tympanic temperature)
- Blood will be drawn for sodium levels
- Blood will be drawn for PK measurements
- Urine and blood will be collected for testing of oxidative stress biomarkers
- Additional urine and blood will be collected for testing osmolality
- ^{31}P MRS: See Attachment 3 for details of the procedures
- OPS: See Attachment 3 for details of the procedures
- Hand fatigue test: See Attachment 3 for details of the procedures
- AEs will be recorded

6.2.1.2.3. Hour 6; 4 Hours after Infusion End and 6 Hours after the Start of Infusion

- Vital signs (heart rate and BP after being supine for 5 minutes, and oral or tympanic temperature)
- Blood will be drawn for sodium levels
- Blood will be drawn for PK measurements
- AEs will be recorded

6.2.1.2.4. Hour 10; 8 Hours after Infusion End and 10 Hours after the Start of Infusion

- Vital signs (heart rate and BP after being supine for 5 minutes, and oral or tympanic temperature)
- ECG (all scheduled ECGs must be performed after the subject has rested quietly for at least 5 minutes in the supine position)
- Hematology analyses (hemoglobin, hematocrit, erythrocyte count [RBC], mean cell volume, mean cell hemoglobin concentration, leukocytes [WBC], neutrophils, lymphocytes, monocytes, eosinophils, basophils, platelets)
- Chemistry analyses (sodium, potassium, total bilirubin, direct bilirubin, alkaline phosphatase, alanine aminotransferase [ALT], aspartate aminotransferase [AST], blood urea nitrogen [BUN], creatinine, uric acid, calcium, nonfasting glucose, albumin, cholesterol, creatine kinase [CK])
- Blood will be drawn for PK measurements
- Urine and blood will be collected for testing of oxidative stress biomarkers
- Additional urine and blood will be collected for testing osmolality
- AEs will be recorded
- After review of vital signs, ECG and laboratory results, subjects may be discharged from the clinical research unit. Note that, if in the opinion of the investigator it is safe and appropriate to discharge the patient prior to review of the data from the 10 hour time point, he/she may do so at his/her discretion.

6.2.1.3. Post-treatment Visit (Visit 3/Day 3)

- Prior and concomitant medications will be recorded and excluded medications will be reviewed
- Vital signs (heart rate and BP after being supine for 5 minutes, and oral or tympanic temperature)
- Hematology analyses (hemoglobin, hematocrit, erythrocyte count [RBC], mean cell volume, mean cell hemoglobin concentration, leukocytes [WBC], neutrophils, lymphocytes, monocytes, eosinophils, basophils, platelets)
- Chemistry analyses (sodium, potassium, total bilirubin, direct bilirubin, alkaline phosphatase, alanine aminotransferase [ALT], aspartate aminotransferase [AST], blood urea nitrogen [BUN], creatinine, uric acid, calcium, nonfasting glucose, albumin, cholesterol, creatine kinase [CK])
- Urine and blood will be collected for testing of oxidative stress biomarkers
- Sustained hand fatigue test: See Attachment 3 for details of the procedures
- AEs will be recorded

6.2.1.4. End of Study Visit (Visit 4/Day 7 [± 1 Day])

- Prior and concomitant medications will be recorded and excluded medications will be reviewed
- Physical examination (general appearance, skin, head, eyes, ears, nose, throat, neck [including thyroid], lymph nodes, chest, heart, abdomen, extremities, and nervous system)
- Weight
- Vital signs (heart rate and BP after being supine for 5 minutes, and oral or tympanic temperature)
- Hematology analyses (hemoglobin, hematocrit, erythrocyte count [RBC], mean cell volume, mean cell hemoglobin concentration, leukocytes [WBC], neutrophils, lymphocytes, monocytes, eosinophils, basophils, platelets)
- Chemistry analyses (sodium, potassium, total bilirubin, direct bilirubin, alkaline phosphatase, alanine aminotransferase [ALT], aspartate aminotransferase [AST], blood urea nitrogen [BUN], creatinine, uric acid, calcium, nonfasting glucose, albumin, cholesterol, creatine kinase [CK])
- Urinalysis (specific gravity, pH, protein, glucose, ketones, occult blood, urine leukocyte esterase)
- Additional urine will be collected for testing of oxidative stress biomarkers
- ^{31}P MRS: See Attachment 3 for details of the procedures
- OPS: See Attachment 3 for details of the procedures
- Hand fatigue test: See Attachment 3 for details of the procedures
- AEs will be recorded

6.2.1.5. Early Discontinuation Visit

- Prior and concomitant medications will be recorded and excluded medications will be reviewed
- Physical examination (general appearance, skin, head, eyes, ears, nose, throat, neck [including thyroid], lymph nodes, chest, heart, abdomen, extremities, and nervous system)
- Weight
- Vital signs (heart rate and BP after being supine for 5 minutes, and oral or tympanic temperature)
- ECG (all scheduled ECGs must be performed after the subject has rested quietly for at least 5 minutes in the supine position)
- Hematology analyses (hemoglobin, hematocrit, erythrocyte count [RBC], mean cell volume, mean cell hemoglobin concentration, leukocytes [WBC], neutrophils, lymphocytes, monocytes, eosinophils, basophils, platelets)
- Chemistry analyses (sodium, potassium, total bilirubin, direct bilirubin, alkaline phosphatase, alanine aminotransferase [ALT], aspartate aminotransferase [AST], blood urea nitrogen [BUN], creatinine, uric acid, calcium, nonfasting glucose, albumin, cholesterol, creatine kinase [CK])
- Urinalysis (specific gravity, pH, protein, glucose, ketones, occult blood, urine leukocyte esterase)
- AEs will be recorded

7. STUDY POPULATION

The inclusion and exclusion criteria for participation in this study are provided below. All screening procedures must be completed during the screening period, but may be performed on different days. Screening procedures cannot be repeated, and subjects cannot be rescreened without the Sponsor's approval. Subjects may only be randomized into the study one time.

7.1. Inclusion Criteria

Subjects are eligible to be included in the study only if they meet all of the following criteria:

1. Are aged ≥60 and ≤85 years
2. Female subjects must be post-menopausal
    a. Post-menopausal women include women with either
        i. Spontaneous amenorrhea for at least 12 months, not induced by a medical condition such as anorexia nervosa and not taking medications during the amenorrhea that induced the amenorrhea (for example, oral contraceptives, hormones, gonadotropin releasing hormone, antiestrogens, selective estrogen receptor modulators, or chemotherapy)

        OR

        ii. Spontaneous amenorrhea for 6 to 12 months and a follicle-stimulating hormone level greater than 40 mIU/mL
3. Have *in vivo* ^{31}P MRS and OPS determined $ATP_{max} < 0.70$ mM/sec
4. Have *in vivo* ^{31}P MRS and OPS determined $P/O < 1.9$
5. Are ambulatory and able to perform activities of daily living without assistance
6. Have sufficient venous access for study drug administration and clinical testing
7. Speak and read English fluently
8. Provide informed consent

7.2. Exclusion Criteria

Subjects will be excluded from the study if they meet any of the following criteria:

1. Have significant disease(s) or condition(s) which, in the opinion of the investigator, may put the subject at risk because of their participation in the study or may influence either the results of the study or the subject's ability to participate in the study
2. Have a history of rhabdomyolysis
3. Have been hospitalized within 3 months prior to screening for major atherosclerotic events (e.g., myocardial infarction, target-vessel revascularization, coronary bypass surgery or stroke) or other major medical condition (as deemed by the primary investigator)
4. Have any metal implants that cannot be removed from the body that in the opinion of the investigator are a contra-indication for undergoing the MRS procedure or any other protocol-related procedure

5. Have an implanted cardiac pacemaker or other implanted cardiac device
6. Have a serum sodium level < 136 mEq/L at Screening or Pre-infusion
7. Have a hemoglobin level < 12 g/dL at Screening or Pre-infusion
8. Have chronic, uncontrolled hypertension as judged by the Investigator (e.g., Baseline systolic blood pressure [SBP] > 140 mm Hg, diastolic blood pressure [DBP] > 90 mm Hg) or a SBP > 150 mm Hg or DBP > 95 mm Hg at the time of Screening or Baseline (if the initial BP reading is above these values, the reading may be repeated one time within 20 minutes of the initial reading).
9. Have a BMI of < 16 or > 35 kg/m^2
10. Have a creatinine clearance < 45 mL/min as calculated by the Cockcroft Gault equation
11. Have a 12-lead ECG demonstrating severe bradycardia (heart rate < 40 bpm) or average QTc > 450 ms for males and > 470 ms for females, and in the opinion of the investigator is clinically significant. (If on the initial ECG, QTc exceeds 450 ms for males or 470 ms for females, the ECG will be repeated 2 more times and the average of the 3 QTc values will be used to determine the subject's eligibility)
12. Have a neurologic disorder that in the opinion of the investigator is a contra-indication for enrollment into the study
13. Have any symptoms consistent with or a current diagnosis of peripheral neuropathy, such as numbness, tingling, pain or altered sensation of hands or feet
14. Have an active, systemic autoimmune disease other than autoimmune thyroid disease (e.g., diabetes, lupus, rheumatoid arthritis) that currently requires treatment or is likely to require treatment during the study.
15. Subject's right hand has history of mobility impairment, fractures, arthritis, hand surgery, muscle disease or other injury that may interfere with any study procedure
16. Have any symptoms consistent with or a current diagnosis of claustrophobia
17. Have a history of cancer, unless subject has documentation of completed curative treatment
18. Have a history of or risk factors (e.g., significant family history, concomitant medical condition) for deep vein thrombosis or pulmonary embolism
19. Have a history of serious mental illness as judged by the Investigator
20. Have a body temperature > 37.5°C at the time of planned dosing
21. Subjects who in the opinion of the investigator abuse alcohol or drugs
22. Have donated or received blood or blood products within the past 30 days
23. Investigator site personnel directly affiliated with this study and/or their immediate families. Immediate family is defined as a spouse, parent, child, or sibling, whether biological or legally adopted.
24. Sponsor employees and/or their immediate families. Immediate family is defined as a spouse, parent, child, or sibling, whether biological or legally adopted.
25. Are currently enrolled in a clinical study involving an investigational product or non-approved use of a drug or device or concurrently enrolled in any other type of medical research judged to be scientifically or medically incompatible with this study

26. Have participated, within the last 30 days in a clinical study involving an investigational product. If the previous investigational product has a long half-life, 3 months or 5 half-lives (whichever is longer) should have passed
27. Have previously been randomized into any study investigating MTP-131 or been exposed to MTP-131 for any reason

7.3. Prohibited Medications

The following medications are prohibited during the study:

- Anti-seizure medications
- Coenzyme Q10, creatine, L-carnitine, and other supplements intended to increase muscle mass, energy or function
- Idebenone
- Muscle relaxants
- Systemic steroid or immunosuppressive use
- Opioids (regular use of > 10 mg/day morphine equivalents)

All other medications, including over-the-counter treatments, must have been unchanged and constant for at least one month prior to the randomization visit and must remain stable until the End-of-Study Visit.

If a subject is on a standing, stable dose of an NSAID at the time of screening, the medication and daily dosage should be reviewed by the investigator as to whether the subject can be safely and appropriately enroll in the study.

When needed, subjects are permitted to take acetaminophen and ibuprofen with consent from the Investigator.

7.4. Discontinuations

7.4.1. Discontinuation of Subjects

The criteria for enrollment must be followed explicitly. If the Investigator identifies a subject who did not meet enrollment criteria and who was inadvertently enrolled, the Sponsor must be notified. If the Sponsor identifies a subject who did not meet enrollment criteria and who was inadvertently enrolled, the Investigator site will be notified. A discussion must occur between the Sponsor and the Investigator to determine whether the subject may continue in the study. An inadvertently enrolled subject may be maintained in the study when the Sponsor agrees with the Investigator that it is medically appropriate for that subject to continue in the study. The Investigator must obtain documented approval from the Sponsor to allow the inadvertently enrolled subject to continue in the study.

Subjects may be discontinued for the following reasons:

- Investigator Decision
    - The Investigator decides that the subject should be discontinued from the study for any reason
    - The investigator determines the subject, for any reason, requires treatment with another therapeutic agent that may affect the efficacy outcomes of this study or is prohibited by this study protocol
- Subject Decision
    - The subject or the subject's designee requests to be withdrawn from the study. No further assessments will be performed, unless the subject provides written consent for follow-up data assessments to be collected.
    - The subject is lost to follow-up: after a reasonable number of attempts to contact the subject, contact or a scheduled assessment fails.
- Sponsor Decision
    - The Sponsor or its designee stops the study or stops the subject's participation in the study for medical, safety, regulatory, or other reasons consistent with applicable laws, regulations, and GCP guidelines.
- Adverse Event
    - If the Investigator decides that the subject should be withdrawn because of an AE or a clinically significant abnormal laboratory value, the investigational product is to be discontinued and appropriate measures are to be taken. The Sponsor or its designee is to be alerted immediately. Any subject withdrawn from the study will be followed until the AE resolves and the subject will complete the Day 7 evaluations.

Every effort will be made to ensure that subjects who discontinue MTP-131 and/or study early will have end-of-study procedures performed as shown in the Study Schedule (Attachment 1).

7.4.2. Discontinuation of Study Sites

Study site participation may be discontinued if the Sponsor or designee, the Investigator, or the ethical review board (ERB) of the study site judges it necessary for medical, safety, regulatory, or other reasons consistent with applicable laws, regulations, and GCP guidelines.

7.4.3. Discontinuation of the Study

The study will be discontinued if the Sponsor or designee judges it necessary for medical, safety, regulatory, or other reasons consistent with applicable laws, regulations, and GCP guidelines.

8. TREATMENT

8.1. Treatments Administered

This study involves a comparison of MTP-131 0.25 mg/kg/hour versus Placebo, both administered intravenously at a rate of 60 mL/hour for 2 hours. The dose of study drug to be administered to each subject may be calculated by the clinical site using the weight measurement from either the Screening or Baseline Visit.

The Investigator or his/her designee is responsible for the following:

- Explaining the correct use of the investigational medicinal product (IMP) to the site personnel
- Verifying that instructions are followed properly
- Maintaining accurate records of IMP dispensing and collection
- Returning all unused IMP to the Sponsor or its designee at the end of the study

Note: In some cases, sites may destroy the material if, during the Investigator site selection, the evaluator has verified and documented that the site has appropriate facilities and written procedures to dispose of clinical study materials.

Subjects will be instructed to contact the Investigator as soon as possible if there is a complaint or problem with the IMP so that the situation can be assessed.

8.2. Materials and Supplies

Study drug will be prepared and stored according to the Pharmacy Manual.

8.2.1. MTP-131

MTP-131 drug product will be provided as a lyophilized powder (20 mg) in sterile glass vials. The lyophilized vials should be maintained in a temperature-monitored refrigerator at 2 to 8°C in a secure area.

Each vial will be aseptically reconstituted with 0.9% sterile saline, by the pharmacy personnel at the site and properly labeled.

8.2.2. Placebo

Placebo matching the appearance of MTP-131 consisting of the powdered lyophilized excipients used for the lyophilized study drug, without the MTP-131 active drug substance, in sterile glass vials. The lyophilized vials should be maintained in a temperature-monitored refrigerator 2 to 8°C in a secure area.

Each vial will be aseptically reconstituted with 0.9% sterile saline, by the pharmacy personnel at the site and properly labeled.

8.3. Method of Assignment to Treatment

Subjects who meet all criteria for enrollment will be randomized 1:1 to double-blind treatment at Visit 2. Assignment to treatment groups will be determined by a computer-generated random sequence using an Interactive Web-Response System (IWRS). The IWRS will be used to assign glass vials containing double-blind investigational product to each subject.

8.4. Selection and Timing of Doses

MTP-131 has been well-tolerated in both clinical and nonclinical studies to date. In nonclinical studies in rats, no significant toxicities were observed at a dose of 3 mg/kg/hour infused continuously for 12 hours. In previous clinical studies, dosing of up to 0.25 mg/kg/hour for 4 hours (total dose of 1 mg/kg) was safe and well-tolerated. The total dose to be administered in this study (0.5 mg/kg) represents approximately half of the highest dose that has been safely administered in previous human studies.

8.5. Continued Access to Investigational Product

MTP-131 will not be made available to study subjects after conclusion of the study.

8.6. Emergency Unblinding

Unblinding the subject allocation to treatment for the purposes of treating AEs is performed through the IWRS, which may supplement or take the place of emergency codes generated by a computer drug-labeling system. This option may be used ONLY if the subject's well-being requires knowledge of the subject's treatment assignment. All instances of unblinding will be recorded and reported by the IWRS.

If an Investigator, site personnel performing assessments, or subject is unblinded, the subject must be discontinued from the study. In cases where there are ethical reasons to have the subject remain in the study, the Investigator must obtain specific approval from the Sponsor for the subject to continue in the study.

In case of an emergency, the Investigator has the sole responsibility for determining if unblinding of a subject's treatment assignment is warranted. Subject safety must always be the first consideration in making such a determination. If the Investigator decides that unblinding is warranted, the Investigator should make every effort to contact the Sponsor prior to unblinding a subject's treatment assignment. If a subject's treatment assignment is unblinded, the Sponsor must be notified immediately.

Upon completion of the study, all codes must be returned to the Sponsor or its designee.

8.7. Concomitant Therapy

All excluded medications and procedures are provided in Section 7.2 and Section 7.3.

8.8. Treatment Compliance

MTP-131 will be administered as an IV infusion and start and completion times will be recorded. Following study completion and unblinding of the study data, MTP-131 blood levels will be determined to confirm that the subject received the appropriate dose of randomized drug.

9. EFFICACY, SAFETY EVALUATIONS, SAMPLE COLLECTION AND TESTING, AND APPROPRIATENESS OF MEASUREMENTS

Study procedures and their timing (including tolerance limits for timing) are summarized in Section 6.2.1 and in the Study Schedule (Attachment 1).

9.1. Efficacy Measures

Skeletal Muscle Energetics and Muscle Performance together comprise Skeletal Muscle Function measures.

9.1.1. Skeletal Muscle Energetics

Changes from Baseline in mitochondrial hand skeletal muscle energetics will be evaluated by the combination 31P MRS/OPS measurements of the following parameters:

- ATP_{max} (phosphorylation capacity per unit muscle volume)
- P/O
- Nicotine adenine dinucleotide (NAD) levels

Details of these testing procedures have been previously published (Jubrias SA et al., 2003). See Attachment 3 for details of the procedures.

9.1.2. Muscle Performance

Hand skeletal muscle performance will be evaluated by changes from Baseline in maximal integrated force generation and muscle work rate calculated from the results of the hand muscle fatigue testing

Details of these testing procedures have been previously published (Jubrias SA et al., 2003). See Attachment 3 for details of the procedures.

9.2. Safety Evaluations

Investigators are responsible for monitoring the safety of subjects who have entered this study and for alerting the Sponsor or designee to any event that seems unusual, even if the event may be considered an unanticipated benefit to the subject. The Investigator is responsible for the appropriate medical care of subjects during the study. The Investigator remains responsible for following, through an appropriate health care option: AEs that are serious, considered related to the study treatment or the study, or caused the subject to discontinue before completing the study.

The safety profile of MTP-131 will be assessed through the recording, reporting, and analyzing of AEs, clinical evaluations, and laboratory tests.

Comprehensive assessment of any apparent toxicity experienced by study subjects will be performed throughout the course of the study, from the time of the subject's signature of informed consent. Study site personnel will report any AE, whether observed by the Investigator or reported by the subject. The reporting period for AEs is described in Section 9.4.6.

9.2.1. *Adverse Events*

An AE is defined as any untoward medical occurrence in the form of signs, symptoms, abnormal laboratory findings, or diseases that emerge or worsen relative to Baseline during administration of an IMP, regardless of causal relationship.

Adverse Events may include the following:

- Suspected adverse drug reactions: side effects known or suspected to be caused by the IMP
- Other medical experiences, regardless of their relationship with the IMP, such as injury, surgery, accidents, extensions of symptoms or apparently unrelated illnesses, and significant abnormalities in clinical laboratory values, psychological testing, or physical examination findings
- Events occurring as a result of protocol interventions (pre- or post-IMP administration)
- Reactions from IMP overdose, abuse, withdrawal, sensitivity, or toxicity.

9.3. Pre-Treatment Events

Untoward events and/or incidental diagnoses that occur prior to IMP administration are by definition, unrelated to the study drug. Pre-treatment events or incidental diagnoses will be recorded on the past medical history eCRF. However, if a pre-treatment event is assessed by the investigator as related to a study procedure and/or meets seriousness criteria, it will be recorded as an AE on the AE eCRF, processed, and followed accordingly.

9.4. Baseline Medical Conditions

Those medical conditions related to the disease under study whose changes during the study are consistent with natural disease progression, or which are attributable to a lack of clinical efficacy of the IMP, are NOT considered as AEs and should not be recorded as such in the eCRF. These are handled in the efficacy assessments and should be documented on the medical history page of the eCRF.

Baseline medical conditions, not in the therapeutic area of interest/investigation, that worsen in severity or frequency during the study should be recorded and reported as AEs.

9.4.1. *Abnormal Laboratory and Other Abnormal Investigational Findings*

Abnormal laboratory findings and other objective measurements should NOT be routinely captured and reported as AEs. However, abnormal laboratory findings or other objective measurements should be reported on the AE pages of the eCRF that:

- meet the criteria for a SAE
- result in discontinuation of the Investigational Medicinal Product
- require medical intervention or
- are judged by the investigator to be clinically significant changes from Baseline

When reporting an abnormal laboratory finding on the AE pages of the eCRF, if available, a clinical diagnosis should be recorded rather than the abnormal value itself (for example, "anemia" rather than "decreased red blood cell count" or "hemoglobin = 10.5 g/dL").

9.4.2. Serious Adverse Events

A SAE is any AE that:

- Results in death. In case of a death, the cause of death is used as the AE term, and the fatality is considered as the OUTCOME.
- Is life-threatening. The term "life-threatening" refers to a SAE in which the subject is at risk of death at the time of the event; it does not refer to an event that hypothetically might cause death if it were more severe.
- Requires inpatient hospitalization or prolongation of existing hospitalization.
- Results in persistent or significant disability or incapacity.
- Is a congenital anomaly or birth defect.
- Is otherwise medically important: Important medical events may be considered as SAEs when, based upon medical judgment, they may jeopardize the subject or may require medical or surgical intervention to prevent one of the outcomes listed in this definition.

For the purposes of reporting, any suspected transmission of an infectious agent via an IMP is also considered an SAE and all such cases should be reported in an expedited manner as described in Section 9.4.7.

9.4.3. Events that Do Not Meet the Definition of a Serious Adverse Event

Elective hospitalizations to simplify study treatment or study procedures (e.g., an overnight stay to facilitate chemotherapy and related hydration therapy application) are not considered as SAEs. However, all events leading to unplanned hospitalizations (not documented prior to ICF signing) or unplanned prolongation of an elective hospitalization (e.g., undesirable effects of any administered treatment) must be documented and reported as SAEs.

9.4.4. Recording of Adverse Events

For the duration of the reporting period, complete, accurate, and consistent data on all AEs experienced will be recorded on an ongoing basis in the appropriate section of the eCRF. In addition, these AEs, all SAEs must be documented and reported using study specific SAE eCRFs as described in Section 9.4.2.

It is important that each AE report include a description of the event along with the duration (onset and resolution dates), severity, relationship to IMP, potential causal/confounding factors, treatment given or other action taken (including dose modification or discontinuation of the IMP), and the outcome.

As the quality and precision of acquired AE data are critical, Investigators should use the AE definitions provided and should observe the following guidelines when completing the AE pages of the eCRF:

- Whenever possible, recognized medical terms should be used to describe AEs rather than lay terms (for example, "influenza" rather than "flu"), and abbreviations should be avoided.
- Adverse events should be described using a specific clinical diagnosis, if available, rather than a list of signs or symptoms (for example, "congestive heart failure" rather than "dyspnea, rales, and cyanosis"). However, signs and symptoms that are not associated with an identified disease or syndrome, or for which an overall diagnosis is not yet available, should be reported as individual AEs.
- Provisional diagnoses (e.g., "suspected myocardial infarction") are acceptable, but should be followed up with a definitive diagnosis if later available. Similarly, a fatal event with an unknown cause should be recorded as "death of unknown cause."
- In cases of surgical or diagnostic procedures, the condition or illness leading to the procedure is considered the AE rather than the procedure itself.

Adverse events occurring secondary to other events (e.g., sequelae or complications) should be identified by the primary cause. A primary AE, if clearly identifiable, generally represents the most accurate clinical term to record in the eCRF.

9.4.5. Investigator Assessments

9.4.5.1. Severity/Intensity

Investigators must assess the severity/intensity of AEs according to the following qualitative toxicity scale:

Mild: The subject is aware of the event or symptom, but the event or symptom is easily tolerated.

Moderate: The subject experiences sufficient discomfort to interfere with or reduce his or her usual level of activity.

Severe: Significant impairment of functioning: the subject is unable to carry out usual activities.

9.4.5.2. Relationship to the Investigational Medicinal Product

Investigators must systematically assess the causal relationship of AEs to the IMP using the following definitions (the decisive factor being the temporal relationship between the AE and administration of the IMP):

Probable: A causal relationship is clinically/biologically highly plausible, there is a plausible time sequence between onset of the AE and administration of the IMP, and there is a reasonable response on withdrawal.

Possible: A causal relationship is clinically/biologically plausible and there is a plausible time sequence between onset of the AE and administration of the IMP.

Unlikely: A causal relationship is improbable and another documented cause of the AE is most plausible.

Unrelated: A causal relationship can be excluded and another documented cause of the AE is most plausible.

9.4.6. Adverse Event Reporting Period

The AE reporting period begins after the first dose of study drug and continues through the clinical study's post-treatment follow-up period defined as 30 days after last administration of study drug. Within a study, all subjects who took at least 1 dose of IMP - whether they completed the treatment period or not - should enter the 30-day safety follow-up period as defined above. If a subject is documented as lost-to follow-up, ongoing/unknown outcome AEs will not be followed-up.

Note: Adverse events that occur between the time subject signs the informed consent form and the time the subject is dosed with study drug will be summarized in the medical history eCRF and not as an AE unless the event meets the definition of an SAE.

Protocol-related AEs (caused by any intervention required by the protocol) and updates on all AEs ongoing or with an unknown outcome must be recorded until the last subject visit required by the protocol. Queries will be sent after the last study visit if ongoing/unknown outcomes of reported AEs are pending. SAEs and medically relevant ongoing/unknown outcome AEs will be followed-up until resolution or stabilization by the Sponsor's Pharmacovigilance department.

Beyond the defined reporting period, any new unsolicited SAE spontaneously reported to the Sponsor by the Investigator will be collected and processed. This and any additional information on SAEs obtained after database lock will reside solely in the Pharmacovigilance study file.

9.4.7. Serious Adverse Event Expedited Reporting

In the event an SAE occurs during the reporting period, the Investigator must immediately (eg, within a maximum of 24 hours after becoming aware of the event) inform the Sponsor as detailed in the Clinical Trial Pharmacovigilance Procedural Manual.

For any SAE, the following minimum information is required as initial notification:

- Investigator/Reporter with full contact information
- Subject identification details (study number, site number, subject number)
- IMP administration details (dose and dates)
- Event verbatim terms, a brief description of signs/symptoms/diagnosis and the date of onset
- Seriousness criteria(ion) met

- Relationship of the event to the IMP (eg, the causality according to the Investigator)

Reporting procedures and timelines are the same for any new follow-up information on a previously reported SAE.

All SAE reports must be completed as described in the eCRF completion guidelines and submitted to the Drug Safety through the Electronic Data Capture (EDC) system of the clinical database. Other relevant information from the clinical database (including demographic data, medical history, concomitant medication, and study drug dosing information) will automatically be sent to the Sponsor's safety department via the EDC system when the SAE form is submitted.

The names, addresses, and telephone and fax numbers for SAE back-up reporting (paper), are included in the Clinical Trial Pharmacovigilance Procedural Manual.

The Investigator/Reporter must respond to any request by the Sponsor for follow-up information (eg, additional information, outcome and final evaluation, specific records where needed) or to any question the Sponsor may have on the SAE within the same timelines as described for initial reports. This is necessary to enable a prompt assessment of the event by the Sponsor and (as applicable) to allow the Sponsor to meet strict regulatory timelines associated with expedited safety reporting obligations.

9.4.8. Pregnancy and In Utero Drug Exposure

Only pregnancies considered by the Investigator as related to study treatment (e.g., resulting from an interaction between study drug and a contraceptive medication) are considered AEs unto themselves. However, all pregnancies with an estimated conception date that occurred during the AE reporting period, as defined in Section 9.4.6 , must be recorded in the AE section of the eCRF. For this study, this applies to pregnancies in female subjects and in female partners of male subjects.

The Investigator must notify the Sponsor in an expedited manner of any pregnancy using the Pregnancy Form and the back-up reporting procedure as described in the Clinical Trial Pharmacovigilance Procedural Manual. Investigators must actively follow-up, document, and report on the outcome of all pregnancies.

The Investigator must notify the Sponsor of these outcomes using Section II of the Pregnancy Form and submit the information using the back-up reporting procedure. Any abnormal outcome must be reported in an expedited manner as described in Section 9.4.7, while normal outcomes must be reported within 45 days from delivery.

In the case of an abnormal outcome, whereby the mother sustains an event, the SAE Report Form is required and will be submitted as described above.

9.4.9. Responsibilities to Regulatory Authorities, Investigators, Ethics Committees, and Ethical/Institutional Review Boards

The Sponsor will send appropriate safety notifications to regulatory authorities in accordance with applicable laws and regulations.

The Investigator must comply with any applicable site-specific requirements related to the reporting of SAEs involving subjects to the Ethics Committee and/or Ethical/Institutional Review Board (EC/ERB/IRB) that approved the study.

In accordance with International Conference on Harmonisation of Technical Requirements for Registration of Pharmaceuticals for Human Use (ICH) GCP guidelines, the Sponsor will inform the Investigator of findings that could adversely affect the safety of subjects, impact the conduct of the study, or alter the EC's/IRB's approval/favorable opinion to continue the study. In particular, and in line with respective regulations, the Sponsor will inform the Investigator of AEs that are both serious and unexpected and are considered to be related to the administered product (suspected unexpected serious adverse reactions or SUSARs). The Investigator should place copies of the safety reports in the Investigator site file. Country-specific regulations with regard to safety report notifications to Investigators will be taken into account.

When specifically required by regulations and guidelines, the Sponsor will provide appropriate safety reports directly to the concerned lead IEC/central IRB and will maintain records of these notifications. When direct reporting by the Sponsor is not clearly defined by country- or site- specific regulations, the Investigator will be responsible for promptly notifying the concerned IEC/IRB of any safety reports provided by the Sponsor and or filing copies of all related correspondence in the site file.

9.5. Sample Collection and Testing

Attachment 1, the Study Schedule, lists the schedule for all sample collections and Attachment 2 lists all clinical laboratory tests that will be performed for this study.

9.5.1. Samples for Study Qualification and Health Monitoring

Blood and urine samples will be collected to determine whether subjects meet inclusion and exclusion criteria and to monitor subject health.

Investigators must document their review of each laboratory safety report.

Samples collected for specified laboratory tests will be destroyed within 60 days of receipt of confirmed test results. Tests are run and confirmed promptly whenever scientifically appropriate. When scientific circumstances warrant, however, it is acceptable to retain samples to batch the tests run, or to retain the samples until the end of the study to confirm that the results are valid. Certain samples may be retained for a longer period, if necessary, to comply with applicable laws, regulations, or laboratory certification standards.

9.5.2. Samples for Drug Concentration Measurements

Blood will be obtained for determination of plasma MTP-131 levels at Baseline (within 30 minutes prior to start of infusion) and then at 2, 6, and 10 hours after the start of infusion.

9.6. Appropriateness of Measurements

The measures used to assess safety in this study are consistent with those widely used and generally recognized as reliable, accurate, and relevant.

10. DATA QUALITY ASSURANCE

To ensure accurate, complete, and reliable data, the Sponsor or representatives will do the following:

- Provide instructional material to the study sites, as appropriate
- Provide start-up training to instruct the Investigators and study coordinators. This training will provide instruction on the protocol, completion of the eCRFs, and study procedures
- Make periodic visits to the study site
- Be available for consultation and maintain contact with the study site personnel by mail, email, telephone, and/or fax
- Review and evaluate eCRF data and use standard computer audit checks to detect errors in data collection
- Conduct a quality review of the database

In addition, the Sponsor or its representatives will periodically check a sample of the subject data recorded against source documents maintained at the study site. The study may be audited by the Sponsor or its representatives, and/or regulatory agencies at any time. Investigators will be given notice before an audit occurs.

To ensure the safety of participants in the study, and to ensure accurate, complete, and reliable data, the Investigator will keep records of laboratory tests, clinical notes, and subject medical records in the subject files as original source documents for the study. If requested, the Investigator will provide the Sponsor, applicable regulatory agencies, and applicable ERBs with direct access to original source documents.

10.1. Data Capture System

An electronic data capture system will be used in this study. The site will maintain a separate source for the data entered by the site into the Sponsor-provided electronic data capture system.

Case report form data will be encoded and stored in a clinical study database. Data managed by a central vendor, such as laboratory test data or ECG data, will be stored electronically in the central vendor's database system.

Any data for which paper documentation provided by the subject will serve, as a source document will be identified and documented by each site in that site's study file. Paper documentation provided by the subject may include, for example, a paper diary to collect subject reported outcome measures (for example, a rating scale), a daily dosing schedule, or an event diary.

Data from complaint forms submitted to the Sponsor will be encoded and stored in the global product complaint management system.

11. SAMPLE SIZE AND STATISTICAL METHODS

11.1. Determination of Sample Size

This study is powered to detect a difference in ATP_{max}, between treatment groups.

Previous studies in endurance training in subjects aged 65 to 80 years suggest the mean ATP_{max} of 0.54 mM/sec, with a standard deviation of 0.04 mM/sec at baseline in the pre-training group (Conley et al., 2013). A total of 20 subjects per treatment group will provide for approximately 90% power to detect difference in mean ATP_{max} of 0.063, between treatment groups, assuming that the common standard deviation is 0.06 using a two group t-test with a 0.05 two-sided significance level.

11.2. Statistical and Analytical Plans

11.2.1. General Considerations

This section describes the general approaches planned to analyze the data from this study. Additional details of the planned analyses outlined here will be further described in the Statistical Analysis Plan (SAP).

For this Phase 2a study, no adjustments will be made to alpha levels to account for multiple efficacy measures. Formal statistical tests (where performed) will be 2-sided and tested at the alpha=0.05 level of significance.

In general, categorical variables will be summarized by the count (N) and percentage of subjects (%). Continuous variables will be summarized by the number of non-missing observations (N), mean, standard deviation, median, minimum, and maximum values.

All study data will be displayed in the data listings.

Statistical analysis of this study will be the responsibility of the Sponsor or designee.

Efficacy analyses will be conducted on the ITT population. This set includes all data from all randomized subjects receiving at least 1 dose of the IMP according to the treatment the subjects were assigned. Safety analyses (and other non-efficacy data displays and summarizations) will be conducted on the safety population, which will include all subjects receiving at least 1 dose of the IMP according to the treatment received.

All tests of treatment effects will be conducted at a 2-sided alpha level of 0.05.

Any change to the data analysis methods described in the protocol will require an amendment ONLY if it changes a principal feature of the protocol. Any other change to the data analysis methods described in the protocol, and the justification for making the change, will be described in the SAP. Additional exploratory analyses of the data will be conducted as deemed appropriate.

11.2.2. Subject Disposition

All subjects who discontinue from the study will be identified, and the extent of their participation in the study will be reported. If known, a reason for their discontinuation will be given.

Subject disposition summaries will include the number of subjects randomized and the numbers included in the Safety and ITT populations by treatment group for all subjects. The number and percentage of subjects who complete or discontinue from the study will be summarized by reason for discontinuation for each treatment group.

11.2.3. Subject Characteristics

Subject's age, sex, weight, height, BMI, and other demographic characteristics will be recorded and summarized by treatment group. Medical history will be listed.

11.2.4. Concomitant Therapy

Summaries of all concomitant therapies taken during the course of the study will be presented in tabular form using Anatomical Therapeutic Chemical 3 classification codes and preferred drug name via the World Health Organization Drug Dictionary (WHO-DD) with version to be specified in the Clinical Study Report. All medications will be summarized by treatment group and sorted alphabetically by medication class and medication subclass.

For the summary tables, if a subject has taken a concomitant medication more than once, the subject will be counted only once in the total.

11.2.5. Primary Efficacy Analyses

The primary efficacy endpoint is change in ATP_{max} from Baseline after study drug infusion (Hour 2). Actual values and change from Baseline will be summarized by treatment group. The primary analysis set is the ITT population.

11.2.6. Secondary Efficacy Analyses

Secondary analyses to address the primary objective will include comparisons in change from Baseline between treatment groups for the following hand skeletal muscle energetics and will be analyzed in the same manner as the primary efficacy measures:

- P/O
- NAD

Mean change from Baseline between treatment groups with respect to muscle performance measured as muscle work rate and maximum integrated force generated will be calculated from the results of the hand muscle fatigue test.

Change from Baseline for the above measures will be assessed at Hour 2 (2 hours after the start of infusion or end of infusion) and Day 7, except for muscle performance which will also be assessed at Day 3. Mean change between treatment groups will be compared in an analysis of covariance (ANCOVA) framework, with Baseline as a covariate.

11.2.7. Plasma Concentration Analyses

Plasma samples will be analyzed for MTP-131 concentrations using a validated liquid chromatography/tandem mass spectrometry assay.

Plasma concentrations will be compared to historical drug levels determined in previous human pharmacokinetic studies in order to provide a preliminary assessment of potential changes in the distribution and clearance of MTP-131 in the elderly population. No formal statistics will be used.

11.2.8. Safety Analyses

Safety measurements will include:

- Adverse events
- Vital signs
- Clinical laboratory evaluations
- ECGs

All safety data will be summarized by treatment group. Baseline values for clinical laboratory evaluations, vital signs and ECGs will be defined as the last evaluation performed prior to administration of study drug.

All AEs will be coded to system organ class (SOC) and preferred term (PT) using the Medical Dictionary for Regulatory Activities (MedDRA) coding dictionary (version to be specified in the clinical study report).

11.2.8.1. Adverse Events

All reported AEs will be listed, but only treatment-emergent adverse events (TEAEs) will be summarized.

The incidence of all TEAEs, drug relationship with TEAEs, and severity of TEAE will be summarized by treatment group. In the summary tables, subjects may be counted under multiple SOCs and PTs, but for each SOC and PT, subjects are only counted once. If a subject has the same AE on multiple occasions, the highest severity (severe > moderate > mild) or drug relationship (probable > possibly related > unlikely related > unrelated) recorded for the event will be presented. If severity is missing, subjects will be included as missing (for severity). If drug relationship is missing, subjects will be included in related tables (eg, considered related). Summary tables will be sorted by SOC, then PT.

11.2.8.2. Deaths and Other Serious Adverse Events

Listings will be provided for the following:

- Deaths
- SAEs
- AEs leading to discontinuation of study drug

11.2.8.3. Clinical Laboratory Evaluations

Summary tables for laboratory parameters (including hematology, chemistry, and urinalysis) will include descriptive statistics for values and change from Baseline for all continuous variables for each treatment group.

Subjects with laboratory data outside the normal range will be listed with abnormal values flagged.

Shift tables (eg, tables that show the number of subjects who are low, normal, or high at Baseline versus each post-baseline scheduled assessment, Attachment 1) will be produced.

The number and percentage of subjects with urinalysis results outside the normal range will be presented by endpoint and visit for each treatment group. Shift tables for urinalysis will show the number of subjects who are normal/abnormal at Baseline and normal/abnormal at the end of study.

11.2.8.4. Vital Signs

Vital sign changes from Baseline and shift tables will be summarized by treatment group and visit.

11.2.8.5. Electrocardiogram

Electrocardiogram results (normal versus abnormal) and an assessment of the clinical significance of any abnormalities (in the opinion of the Investigator) will be listed for individual subjects. Intervals of PR, QRS and QTc will also be listed.

11.2.8.6. Other Safety Parameters

Any other safety data captured on the eCRF will be listed.

11.2.9. Interim Analyses

No interim analyses are planned for this study.

12. INFORMED CONSENT, ETHICAL REVIEW, AND REGULATORYCONSIDERATIONS

12.1. Informed Consent

The Investigator is responsible for ensuring that the subject understands the potential risks and benefits of participating in the study, including answering any questions the subject may have throughout the study and sharing in a timely manner any new information that may be relevant to the subject's willingness to continue his or her participation in the study.

The informed consent form (ICF) will be used to explain to the subject the potential risks and benefits of study participation to the subject in simple terms before the subject is entered into the study, and to document that the subject is satisfied with his or her understanding of the risks and benefits of participating in the study and desire to participate in the study.

The Investigator is responsible for ensuring that informed consent is given by each subject or legal representative. This includes obtaining the appropriate signatures and dates on the ICF prior to the performance of any protocol procedures and prior to the administration of investigational product.

12.2. Ethical Review

The Sponsor or its representatives must approve all ICFs before they are used at investigative sites. All ICFs must be compliant with ICH GCP guidelines.

Documentation of ERB approval of the protocol and the ICF must be provided to the Sponsor before the study may begin at the investigative site. The ERB will review the protocol as required.

The study site's ERB should be provided with the following:

- The current IB and updates during the course of the study
- ICF
- Relevant curricula vitae

12.3. Regulatory Considerations

This study will be conducted in accordance with:

- Consensus ethics principles derived from international ethics guidelines, including the Declaration of Helsinki and Council for International Organizations of Medical Sciences (CIOMS) International Ethical Guidelines
- The ICH GCP Guideline [E6]
- Applicable laws and regulations

The Investigator or designee will promptly submit the protocol to applicable ERB(s). Some of the obligations of the Sponsor may be assigned to a third party organization.

An identification code assigned to each subject will be used in lieu of the subject's name to protect the subject's identity when reporting AEs and/or other study-related data.

12.3.1. Protocol Signatures

The Sponsor's responsible medical officer will approve the protocol, confirming that, to the best of his or her knowledge, the protocol accurately describes the planned design and conduct of the study.

After reading the protocol, the Investigator will sign the protocol signature page and send a copy of the signed page to a Sponsor representative.

12.3.2. Final Report Signature

The Investigator will sign the final clinical study report for this study, indicating agreement with the analyses, results, and conclusions of the report.

The Sponsor's responsible medical officer and statistician will approve the final clinical study report for this study, confirming that, to the best of his or her knowledge, the report accurately describes the conduct and results of the study.

12.3.3. Study Monitoring

The Investigators and institution(s) will permit study-related monitoring of the Case Report Form data by Stealth BioTherapeutics Inc., or their assignee by providing direct access to source data and/or documents. The study monitor will verify the eCRFs 100% against the source documentation. Deviations from the protocol with regard to subject enrollment or study conduct will also be noted in the source documentation, in the eCRF and a complementary database. A Sponsor representative will visit the site to initiate the study, prior to the first treatment of the first subject, and at agreed times throughout the study, including at the end of the study. Medication dispensing and clinical drug supply records will be 100% verified at the study site by the study monitor. It is understood that all subject specific information is confidential and no documentation that can link study information to the specific subject will be collected or retained by the sponsor.

12.3.4. Retention of Records

All study related material including source documents, eCRFs, CA and EC correspondence and analyses and any other documentation required by applicable laws and regulations will be maintained for fifteen (15) years after completion of the study or notification from the Sponsor that the data can be destroyed, whichever comes first.

12.3.5. Disclosure of Information

The Investigator may use this information for the purposes of the study only. It is understood by the Investigator that Stealth BioTherapeutics Inc. will use information developed in this clinical study in connection with the development of the investigational medication and therefore may disclose it as required to other clinical investigators and to regulatory agencies. In order to allow the use of the information derived from this clinical study, the Investigator understands that he/she has an obligation to provide complete test results and all data developed during this study to the Sponsor.

The Investigator may not submit for publication or presentation the results of this study without first receiving written authorization from Stealth BioTherapeutics Inc. Stealth BioTherapeutics Inc. agrees that, before it publishes any results of the study, it shall provide the Investigator with at least 30 days for review of the pre-publication manuscript prior to the submission of the manuscript to the publisher.

13. REFERENCES

Bortz WM 2nd. A Conceptual Framework of Frailty: a Review. *J Gerontol A Biol Sci Med Sci*. 2002;57A(5):M283-M288.

Conley KE, Jubrias SA, Cress ME, Esselman PC. Elevated energy coupling and aerobic capacity improves exercise performance in endurance-trained elderly subjects. *Exp Physiol.* 2013 Apr;98(4):899-907.

Janssen I, Shepard DS, Katzmarzyk PT, Roubenoff R. The Healthcare Costs of Sarcopenia in the United States. *J Am Geriatr Soc.* 2004;52(1):80-85.

Jubrias SA et. al. Acidosis inhibits oxidative phosphorylation in contracting human skeletal muscle in vivo. *J Physiol.* 2003;533(2):589-99.

Jubrias SA et. al. Large energetic adaptations of elderly muscle to resistance and endurance training. *J Appl Physiol.* 2001; 90:1663-1670.

Min K, Smuder AJ, Kwon O, Kavazis AN, Szeto HH, Powers SK. Mitochondrial-targeted Antioxidants Protect Skeletal Muscle Against Immobilization-induced Muscle Atrophy. *J Appl Physiol*. 2011;111(5):1459-1466.

Powers SK, Hudson MB, Nelson WB, Talbert EE, Min K, Szeto HH, Kavazis AN, Smuder AJ. Mitochondria-targeted Antioxidants Protect Against Mechanical Ventilation-induced Diaphragm Weakness. *Crit Care Med*. 2011;39(7):1749-1759.

Righi V, Constantinou C, Mintzopoulos D, Khan N, Mupparaju SP, Rahme LG, Swartz HM, Szeto HH, Tompkins RG, Tzika AA. Mitochondria-targeted Antioxidant Promotes Recovery of Skeletal Muscle Mitochondrial Function after Burn Trauma Assessed by *in vivo* ^{31}P Nuclear Magnetic Resonance and Electron Paramagnetic Resonance Spectroscopy. *FASEB J*. 2013;27(6):2521-2530.

Rondelli RR, Dal Corso S, Simões A, Malaguti C. Methods for the Assessment of Peripheral Muscle Fatigue and Its Energy and Metabolic Determinants in COPD. *J Bras Pneumol*. 2009;35(11):1125-1135.

Siegel MP, Kruse SE, Percival JM, Goh J, White CC, Hopkins HC, Kavanagh TJ, Szeto HH, Rabinovitch PS, Marcinek DJ. Mitochondrial-targeted Peptide Rapidly Improves Mitochondrial Engergetics and Skeletal Muscle Performance in Aged Mice. Aging Cell. 2013;12(5):763-771.

Smart, N. Exercise Training for Heart Failure Patients with and without Systolic Dysfunction: an Evidence-based Analysis of How Patients Benefit. *Cardiol Res Pract*. 2011; doi: 10.4061/2011/837238.

Smart N, Haluska B, Jeffriess L, Marwick TH. Exercise Training in Systolic and Diastolic Dysfunction: Effects on Cardiac Function, Functional Capacity, and Quality of Life. *Am Heart J*. 2007;153(4):530-536.

Williams AD, Carey MF, Selig S, Hayes A, Krum H, Patterson J, Toia D, Hare DL. Circuit Resistance Training in Chronic Heart Failure Improves Skeletal Muscle Mitochondrial ATP Production Rate--a Randomized Controlled Trial. *J Card Fail*. 2007;13(2):79-85.

ATTACHMENT 1. STUDY SCHEDULE

Study Schedule, Protocol SPITM-201

	Visit 1	Visit 2					Visit 3	Visit 4	Early Discontinuation Visit
	Day -28 to -7	Day 1 (Baseline)[a]					Day 3 (-1 day)	Day 7 (±1 day)	
	Screening	Pre-Infusion	Infusion Start (Hour 0)	Hour 2[b]	Hour 6[c]	Hour 10[d]	Post-Treatment Visit	End of Study Visit	
Informed Consent	X								
Admit to Clinical Research Unit		X							
Randomization		X							
Study Drug Administration			X						
Inclusion/ Exclusion Criteria	X	X							
Verify Continued Eligibility		X							
Demographics	X								
Medical and Surgical History	X								
Prior and Concomitant Medications	X	X					X	X	X
Physical Examination and Weight	X	X						X	X
Height[e]	X								
Vital Signs	X	X		X	X	X	X	X	X
Electrocardiogram[f]	X	X				X			X
Hematology[g]	X	X				X	X	X	X
Clinical Chemistry[h]	X	X				X	X	X	X
Sodium level[i]				X	X				
Pharmacokinetic Blood Samples[j]		X		X	X	X			
Urinalysis[k]	X	X						X	X

Study Schedule, Protocol SPITM-201, continued

	Visit 1	Visit 2					Visit 3	Visit 4	Early Discontinuation Visit
	Day -28 to -7	Day 1 (Baseline)[a]					Day 3 (-1 day)	Day 7 (±1 day)	
	Screening	Pre-Infusion	Infusion Start (Hour 0)	Hour 2[b]	Hour 6[c]	Hour 10[d]	Post-Treatment Visit	End of Study Visit	
Oxidative Stress Biomarkers[l]		X		X		X	X	X	
Serum and urine osmolality [m]		X		X		X			
^{31}P Magnetic Resonance Spectroscopy[n]	X			X				X	
Optical Spectroscopy Scan[n]	X			X				X	
Hand Fatigue Test[n]	X			X			X[p]	X	
Adverse Events[o]	X	X	X	X	X	X	X	X	X
Discharge from Clinical Research Unit						X			

a. There must be at least 7 but not more than 28 days between Screening and Randomization/Infusion.

b. Hour 2 = 2 hours after the start of study drug infusion and the time of study drug infusion completion.

c. Hour 6 = 6 hours after the start of study drug infusion and 4 hours after study drug infusion completion.

d. Hour 10 = 10 hours after the start of study drug infusion and 8 hours after study drug infusion completion.

e. Height will be measured at Screening only in order to calculate BMI.

f. ECGs will be obtained at Screening, and Baseline (prior to start of infusion) and 10 hours (± 15 minutes) after the start of study drug infusion. All scheduled ECGs must be performed after the subject has rested quietly for at least 5 minutes in the supine position.

g. Hematology will be obtained at Screening, and Baseline (prior to start of infusion), 10 hours (± 15 minutes) after the start of study drug infusion, at Day 3 and Day 7.

h. Clinical chemistry will be obtained at screening and, Baseline (prior to start of infusion), 10 hours (± 15 minutes) after the start of study drug infusion, at Day 3 and Day 7.

i. Sodium will be obtained at 2 and 6 hours (± 15 minutes) after the start of the study drug infusion.

j. Blood for pharmacokinetic (PK) analysis will be obtained at Baseline (prior to start of infusion) and then at 2, 6, and 10 hours (± 15 minutes) after the start of study drug infusion.

k. Urinalysis will be obtained at Screening, and Baseline (prior to start of infusion) and Day 7.

l. Oxidative Stress Biomarkers consist of serum myostatin and urinary 8-isoprostane, 8-hydroxy-2-deoxyguanosine and nitrotyrosine. Samples should be collected at Baseline (prior to start of infusion) and at 2 and 10 hours (± 30 min) after the start of the infusion and at Day 3 and Day 7.

m. Serum and urine for osmolality will be obtained at Baseline (prior to start of infusion) and then at 2 and 10 hours (± 15 min) after the start of study drug infusion.

n. See Attachment 3 for details. The ^{31}P MRS, OS, and Hand Fatigue testing will occur as a group, and will begin within 30 minutes after completing the study drug infusion. During Visit 2, Subjects may not eat from 1 hour prior to the start of the infusion until completion of the ^{31}P MRS, OPS, and hand fatigue test. Subjects may drink up to 1 liter of water during this time. During Visit 3, Subjects may not eat from 1 hour prior to the Hand Fatigue test until completion of this test. Subjects may drink up to 1 liter of water during this time. During Visit 4, Subjects may not eat from 1 hour prior to the ^{31}P MRS, OPS, and hand fatigue test. Subjects may drink up to 1 liter of water during this time.

o. Adverse events occurring before administration of study drug will be recorded in the medical history eCRF.

p. At Visit 3, only the Sustained Hand Fatigue portion of the test will be administered.

ATTACHMENT 2. CLINICAL LABORATORY TESTS

Clinical Laboratory Tests

**Hematology:**	**Clinical Chemistry (serum concentrations):**
Hemoglobin	Calcium
Hematocrit	Sodium
Erythrocyte count (RBC)	Potassium
Mean RBC volume	Total bilirubin
Mean RBC hemoglobin concentration	Direct bilirubin
Leukocytes (WBC)	Alkaline phosphatase
Neutrophils	Alanine aminotransferase (ALT)
Lymphocytes	Aspartate aminotransferase (AST)
Monocytes	Blood urea nitrogen (BUN)
Eosinophils	Creatinine
Basophils	Uric acid
Platelets	Albumin
Glucose, nonfasting	Cholesterol
**Urinalysis:**	Creatine kinase (CK)
Specific gravity	**Pharmacokinetic Blood Samples:**
pH	Serum and urine for osmolality
Protein	
Glucose	**Oxidative stress biomarkers:**
Ketones	Serum myostatin
Occult blood	Urinary 8-isoprostane
Urine leukocyte esterase	Nitrotyrosine

Abbreviations: RBC = red blood cells; WBC = white blood cells.

ATTACHMENT 3: MAGNETIC RESONANCE SPECTROSCOPY, OPTICAL SPECTROSCOPY, AND MUSCLE FATIGUE TESTING PROTOCOLS

Informed consent for these procedures will be obtained within the main informed consent form for this study. Specific Screening procedures must be used to determine suitability for these procedures; these include blood pressure testing (cuff will be inflated to +50 torr over resting systolic pressure).

The Magnetic Resonance Spectroscopy, Optical Spectroscopy, and Muscle Fatigue Testing Protocols will be performed for each subject as described below:

1. ^{31}P MRS Procedures (total time, approximately 1.5 hours)
    a. Positioning/Scanning (~20 minutes)
        i. Hand Positioning: Fit right hand into magnetic resonance (MR) cradle to position index finger for force measurement, secure MR coil to hand with tape and secure arm/hand to cradle with Velcro, and fit the blood pressure (BP) cuff on upper right arm.
        ii. Subject Positioning: Move subject into magnet room, position subject in supine position on gurney, place MR holder in magnet and adjust subject's position on gurney to ensure comfort.
        iii. Cradle Positioning in Magnet: Adjust position of MR cradle in magnet bore to center of magnet and attach all electronic connections.
        iv. Hand Muscle Force Measure: Request the subject to push index finger against force transducer to generate a muscle maximum voluntary contraction (MVC). Follow force by the number of lights activated on a Light Emitting Diode (LED) panel. Instruct the subject to activate half the number of lights (70% MVC) during the exercise period.
        v. Scanning: Provide subject with earplugs, inform about MR generated noises, tune MR probe, close door, optimize MR measures, and take fully relaxed spectrum.
    b. Ischemia (~40 minutes)
        i. Pre-ischemia Measures: Instruct subject about timing, start experimental scans (5 minutes), and warn subject 1 minute before ischemia/exercise.
        ii. Ischemic Protocol: Start scan of resting first dorsal interosseous (FDI) muscle (Minute 0); warn subject about onset of ischemia (Minute 7); inflate BP cuff or right arm and instruct subject to start exercise (Minute 8); contract muscle with each beat of a metronome (40 beats/minute) until instructed to stop (~30 sec; Minute 8.5), ischemia is maintained until Minute 23 (total ischemia = 15 minutes), release cuff and allow muscle to recover until Minute 35 (total recovery = 12 minutes).

iii. Subject Removal: Detach cables, assist subject in withdrawing from magnet, moving off gurney and returning to lab bench to remove MR coil and cradle, remove BP cuff.

2. Optical Spectroscopy Procedures (~1 hour)

   a. Hand Positioning: Fit right hand into optics cradle with optics probe positioned over FDI muscle, fit the BP cuff on upper right arm, fit subject with mask for 100% O_2.

   b. Optics/Ischemia Measures: Collect optical spectra for 3 minutes, turn on O_2 supply for 5 more minutes and continue throughout protocol; inflate BP cuff (Minute 8) and keep inflated for 15 minutes; release BP cuff at Minute 23 and follow recovery for 12 minutes (to Minute 38).

   c. Subject Removal: Detach optical probe, remove O_2 mask and BP cuff.

3. Muscle Fatigue Procedures (Hand Fatigue; 1 hour)

   a. Exercise Instruction: Review exercise protocol to re-familiarize subject.

      i. ATP_{max} Test: Instruct subject to exercise FDI muscle as fast as possible when instructed and to stop immediately when instructed (~30 seconds)

      ii. Sustained Hand Fatigue Test: Review exercise protocol. Request the subject to push index finger against force transducer to generate a muscle MVC. Instruct subject to exercise FDI muscle at 70% MVC (as shown by the light box) at rate set by a metronome for 2 minutes and to increase rate with a metronome every
      2 minutes. Practice with hand in MR cradle outside magnet. Practice tests with right hand in MR cradle outside magnet.

   b. Positioning/Scanning (~20 minutes)

      i. Hand Positioning: Fit right hand into MRS cradle to position finger for force measurement, secure MR coil to hand with tape and secure arm/hand to cradle with Velcro, and fit the BP cuff on upper right arm.

      ii. Subject Positioning: Move subject into magnet room, position subject in supine position on gurney, place MR holder in magnet and adjust subject's position on gurney to ensure comfort.

      iii. Cradle Positioning in Magnet: Adjust position of MR cradle in magnet bore to center of magnet and attach all electronic connections.

      iv. Hand Muscle Force Measure: Request the subject to push index finger against force transducer to generate a muscle MVC. Follow force by the number of lights activated on a LED panel. Instruct the subject to activate half the number of lights (70% MVC) during the exercise period.

      v. Scanning: Provide subject with earplugs, inform about MR generated noises, tune MR probe, close door, optimize MR measures, and take fully relaxed spectrum.

c. ATP_{max} Test (~20.5 minutes): Start scan of resting FDI muscle (Minute 0); warn subject about onset of exercise (Minute 7); instruct subject to start exercise (Minute 8); contract muscle as fast as possible until instructed to stop (~30 seconds; Minute 8.5), allow muscle to recover until Minute 20.5 (total recovery = 12 minutes).

d. Fatigue Test (~20 minutes): Start scan of resting muscle (Minute 0); warn subject about onset of exercise (Minute 7); instruct subject to start exercise at 70% MVC (Minute 8) at the rate set by a metronome and to increase exercise rate with the faster beat of the metronome, which occurs in 2 minute increments until 70% MVC cannot be maintained (~8 minutes; Minute 16); allow muscle to recover for 12 minutes.

e. Subject Removal: Detach cables, assist subject in withdrawing from magnet, moving off gurney and returning to lab bench to remove MR coil and cradle.
